# Histological and Histomorphometric Human Results of HA-Beta-TCP 30/70 Compared to Three Different Biomaterials in Maxillary Sinus Augmentation at 6 Months: A Preliminary Report

**DOI:** 10.1155/2015/156850

**Published:** 2015-07-26

**Authors:** Susanna Annibali, Giovanna Iezzi, Gian Luca Sfasciotti, Maria Paola Cristalli, Iole Vozza, Carlo Mangano, Gerardo La Monaca, Antonella Polimeni

**Affiliations:** ^1^Department of Oral and Maxillofacial Sciences, Oral Surgery Unit, School of Dentistry, Sapienza University of Rome, Via Caserta 6, 00161 Rome, Italy; ^2^Department of Medical, Oral and Biotechnological Sciences, University of Chieti-Pescara, Chieti, Italy; ^3^Department of Biotechnologies and Medical Surgical Sciences, Sapienza University of Rome, Rome, Italy; ^4^Department of Surgical and Morphological Sciences, University of Insubria, Varese, Italy

## Abstract

*Objective*. The aim of this investigation was to examine the bone regenerative potential of newly biphasic calcium phosphate ceramics (HA-*β*-TCP 30/70), by assessing histological and histomorphometric results of human specimens retrieved from sinuses augmented with HA-*β*-TCP 30/70, and comparing them to anorganic bovine bone (ABB), mineralized solvent-dehydrated bone allograft (MSDBA), and equine bone (EB), after a healing period of 6 months. *Materials and Methods*. Four consecutive patients with edentulous atrophic posterior maxilla were included in this report. A two-stage procedure was carried out for sinus augmentation with HA-*β*-TCP 30/70, ABB, MSDBA, and EB. After 6 months, specimens were retrieved at the time of implant placement and processed for histological and histomorphometric analyses. *Results*. At histological examination, all biomaterials were in close contact with the newly formed bone and showed the same pattern of bone formation; the grafted granules were surrounded by a bridge-like network of newly formed bone. A limited number of ABB particles were partially covered by connective tissue. The histomorphometric analysis revealed 30.2% newly formed bone for Ha-*β*-TCP 30/70, 20.1% for ABB, 16.4% for MSDBA, and 21.9% for EB. *Conclusions*. Within the limitations of the present investigation, these results support the successful use of HA-*β*-TCP 30/70 for sinus augmentation.

## 1. Introduction

Deficiencies in the volume and quality of available bone can complicate implant placement in the resorbed maxillary jaw. Sinus grafting in the maxillary posterior region is a highly predictable procedure to increase the vertical volume of sinus floor bone to accommodate dental implants [1, 2].

Autologous bone is considered the gold standard for bone regeneration [3, 4], even if different bone substitutes have been proposed to overcome the limits related to its use, specifically, donor site morbidity, increased operating time, need of a second surgical site to obtain the transplant, and potential intraoperative and postoperative complications [5–7]. After implant placement, a resorption with up to 49.5% marked volume loss has been reported in the literature after 6 months from sinus grafting [8, 9]. A number of graft materials with different origin and mechanism of bone regeneration have been used alone or in combination with autografts in sinus floor augmentations [10–20]. Therefore the current issue is the definition of the best filling material for the sinus cavity. The presence or absence of autogenous bone in a graft did not affect implant survival [21, 22] and different studies have suggested that some alternative augmentation materials may not adversely influence clinical outcomes and implant survival [23, 24].

Among the graft materials used in maxillary sinus augmentation procedures, biphasic calcium phosphate ceramics, reached by mixing hydroxyapatite (HA) and tricalcium phosphate (TCP), are considered biocompatible, osteoconductive, and suitable to obtain bone formation [24]-[25]. The HA, undergoing slow resorption, works as a scaffold to maintain space, while the TCP that underwent quick dissolution leads to more interparticle space and releases calcium and phosphorus ions that could stimulate new bone formation, promoting osteogenic activity [26]. Currently several bioceramic materials are used and differences in HA/TCP ratio, phase composition, formulation, sizes, and shapes affect their biological and mechanical properties [10, 18], [24], [27–29].

The newly biphasic calcium phosphate ceramics (HA-*β*-TCP 30/70) with reticular structure, tested in the present investigation, seem to have a better resorption and an increased bone formation [18].

Anorganic bovine bone (ABB) is a deproteinized sterilized bovine bone, constituted by a calcium-deficient carbonate apatite, with 75% porosity and a crystal size of about 10 nm in the form of granules. Due to its natural structure, it is chemically and physically comparable to human bone and its porous nature, greatly increasing the surface area of the material, promotes angiogenesis and immigration of osteogenic cells [19]. The usage of ABB is widely documented and shows that it can well integrate in host bone tissue in different clinical and histological results [30–32].

MSDBA is a solvent-dehydrated, limited-dose, gamma-irradiated portions of human iliac crest bone wedge. The allograft mixture contains 50% cortical and 50% cancellous microchips with a particle size of 1-2 mm. MSDBA showed significant histological results in terms of new bone formation after sinus augmentation procedures [10, 11, 33].

Equine-derived bone (EB) is an equine-derived bone tissue deantigenated by a proteolytic low-temperature process that eliminates the organic components but leaves the mineral structure of the hydroxyapatite unaltered, saving the resorption potential [12]. Recent investigations showed that also the equine-derived bone (EB) is able to induce bone formation in maxillary sinus augmentations [12, 13, 34, 35].

Although the number of newly formed bones in the augmented sinus is not directly related to the survival rate of the implants, histological and histomorphometric analysis of specimens, retrieved at the time of implant placement (after a healing period of 6 months), represents a reliable indicator to assess and compare the performance of the graft materials [19].

The aim of this investigation was to examine the bone regenerative potential of a newly biphasic calcium phosphate ceramics (HA-*β*-TCP 30/70), by assessing histological and histomorphometric results of human specimens retrieved from sinuses augmented with HA-*β*-TCP 30/70, and comparing them to anorganic bovine bone (ABB), mineralized solvent-dehydrated bone allograft (MSDBA), and equine bone (EB), after a healing period of 6 months.

## 2. Materials and Methods

### 2.1. Patient Selection

Four healthy patients (3 males, 1 females, all nonsmokers, mean age 52 years, range 36–70 years), scheduled for sinus augmentation in the atrophic posterior maxilla to receive fixed restorations, were recruited for this study. Inclusion criteria were maxillary partial edentulism in the premolar/molar areas and subsinus residual bone height, measured on computerized tomography (CT) scan, ranging from 2 to 4 mm and bone thickness 3–5 mm. Exclusion criteria were smoking, patients with systemic diseases and maxillary sinus pathology. The clinical procedures were performed in accordance with the Declaration of Helsinki and the Good Clinical Practice Guidelines. All patients signed a written informed consent form. After reviewing medical history and making a preliminary clinical examination, digital panoramic radiography and CT scan were performed. A two-stage procedure was carried out for sinus floor augmentation with HA-*β*-TCP 30/70, ABB, MCBA, and EB. These materials were allocated to the participant's sinus under randomized conditions. After a healing period of 6 months, specimens were retrieved at the time of implant placement and processed for histological and histomorphometric analyses.

### 2.2. Surgical Procedure

Surgery was performed under sterile conditions and local anaesthesia with mepivacaine 2% with epinephrine 1 : 100.000 (Carbocaine, AstraZeneca, Italy). On the day of surgery, each patient was administered 2 g amoxicillin + clavulanic acid (Augmentin, GlaxoSmithKline, London, UK) 1 hour prior to surgery and rinsed with a chlorhexidine digluconate solution 0.2% (Corsodyl, GlaxoSmithKline, Belgium) for 2 min immediately prior to the intervention. Sinus floor elevation was performed utilizing a lateral window technique. A slightly palatal crestal incision was made and supplemented by buccal releasing incisions mesially and distally. Full thickness flaps were elevated to expose the alveolar crest and the lateral sinus wall. An oval-shaped osteotomy, with the inferior border about 5 mm superior to the alveolar bone margin, was made on the lateral aspect of the sinus wall using a round bur under cold sterile saline irrigation. The center of the bony window was gently in-fractured with care to ensure that its most superior portion was left intact. The sinus membrane was carefully elevated and rotated, together with the osteotomy window, inward and upward. The exposed sinus cavity was augmented with HA-*β*-TCP 30/70 ABB, MCBA, or EB. The graft was placed and carefully packed into the area between the elevated Schneiderian membrane and sinus floor without excessive pressure. A resorbable membrane was placed over the lateral wall of the sinus and the mucoperiosteal flap was replaced and stabilized with monofilament, nonresorbable, expanded polytetrafluoroethylene (e-PTFE) (Gore-Tex Suture, W. L. Gore, Flagstaff, AZ, USA) sutures. No Schneiderian membranes were perforated during any of the sinus augmentation procedures. For the postoperative phase, the patients were protected from infection by administration of prophylactic antibiotics (Augmentin, GlaxoSmithKline, London, UK) 1 g, 2 times daily for 1 week; an analgesic and antiphlogistic medication (Ibifen, 200 mg -IBI-Lorenzini, Aprilia, Italy) was prescribed 3 times daily. The patient was instructed to avoid use of a removable prosthesis, not to blow the nose, and to rinse twice daily for a period of 2 weeks with 0.2% chlorhexidine gluconate. Following a healing period of 2 weeks, sutures were removed. Six months after the augmentation procedure, clinical and radiographic examination were undertaken and each patient was reappointed for biopsy at the time of implant placement in the same location. Under local anesthesia, a vertical incision was made buccally and continued horizontally and distally at the palatal side of the alveolar crest. A full thickness flap was raised and mobilized for tension-free closure. During this surgery, a total of 4 bone samples were retrieved with a 3.5 trephine bur under sterile saline solution irrigation, one from each augmented site. From 1 to 6 implants were placed into each patient's augmented sinus according to the manufacturer's directions.

### 2.3. Histological Procedure

The bone cores were retrieved and were immediately stored in 10% buffered formalin and processed to obtain thin ground sections. The specimens were processed using the Precise 1 Automated System (Assing, Rome, Italy) [36]. The specimens were dehydrated in a graded series of ethanol rinses and embedded in a glycolmethacrylate resin (Technovit 7200 VLC, Kulzer, Wehrheim, Germany). After polymerization, the specimens were sectioned, along their longitudinal axis, with a high precision diamond disk at about 150 *μ*m and ground down to about 30 *μ*m with a specially designed grinding machine Precise 1 Automated System (Assing, Rome, Italy). Three slides were obtained from each specimen. These slides were stained with acid fuchsin and toluidine blue and examined with transmitted light Leitz Laborlux microscope (Leitz, Wetzlar, Germany). Histomorphometry of the percentages of newly formed bone, residual grafted material, and marrow spaces was carried out using a light microscope (Laborlux S, Leitz, Wetzlar, Germany) connected to a high-resolution video camera (3CCD, JVC KY-F55B, JVC, Yokohama, Japan) and interfaced with a monitor and PC (Intel Pentium III 1200 MMX, Intel, Santa Clara, CA, USA). This optical system was associated with a digitizing pad (Matrix Vision GmbH, Oppenweiler, Germany) and a histometry software package with image capturing capabilities (Image-Pro Plus 4.5, Media Cybernetics Inc., Immagini & Computer Snc, Milano, Italy).

## 3. Results

### 3.1. Clinical Results

In all cases, no perforations of the Schneiderian membrane were present, primary stability of the implants was achieved, regardless of the bone graft substitute used, and postoperative complications were absent. The healing process after sinus augmentation procedure was uneventful and no clinical signs of sinus pathology were observed. Six months after augmentation, the radiographic examination showed in all patients the presence of dense bone in the maxillary sinuses where the biomaterials were inserted.

### 3.2. Histological and Histomorphometric Results

#### 3.2.1. Biphasic Calcium Phosphate Ceramics (HA-*β*-TCP 30/70)

**Figure 1:**
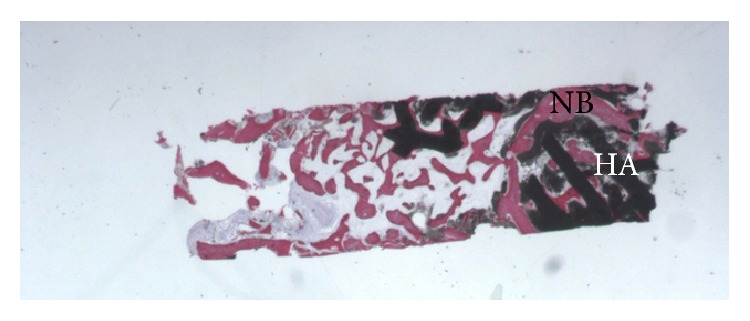
Grafted HA-*β*-TCP 30/70 (HA) particles, almost completely surrounded by newly formed bone (NB) (acid fuchsin-toluidine blue; original magnification 12x).

**Figure 2:**
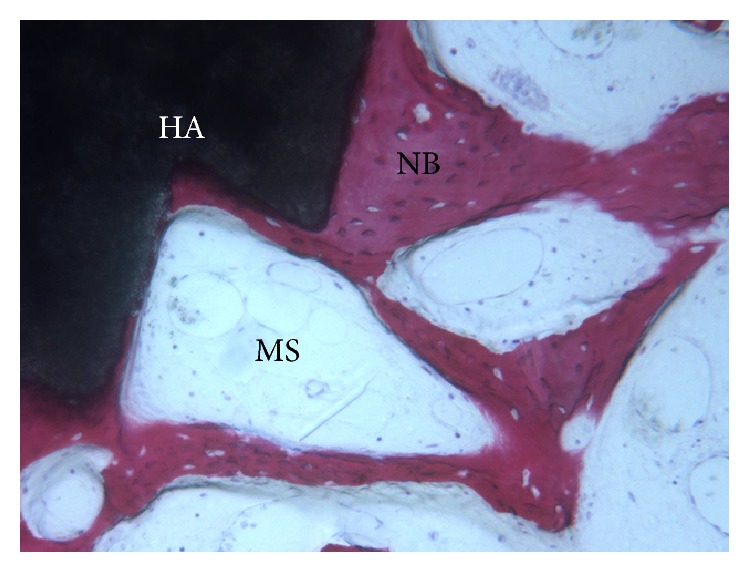
Marrow stromal cells (MS) and blood vessels were found in the vicinity of newly formed bone (NB) surrounding HA-*β*-TCP 30/70 particles (HA) (acid fuchsin-toluidine blue; original magnification 100x).

At low magnification, a large number of grafted biomaterials, completely surrounded by newly formed bone, were observed (Figure 1). In some fields osteoblasts were detected in the process of apposing bone directly on the particle surface. No gaps were present at the bone-particles interface, and the bone was always in close contact with the particles. Marrow stromal cells and blood vessels were found inside the marrow spaces. A vascular growth was also observed next to the newly formed bone (Figure 2). No inflammatory cells or foreign body reaction was noted around the grafted particles. Histomorphometry showed that newly formed bone represented 30.2%, marrow spaces 40.7%, and the residual graft material 29.1%.

#### 3.2.2. Anorganic Bovine Bone (ABB)

**Figure 3:**
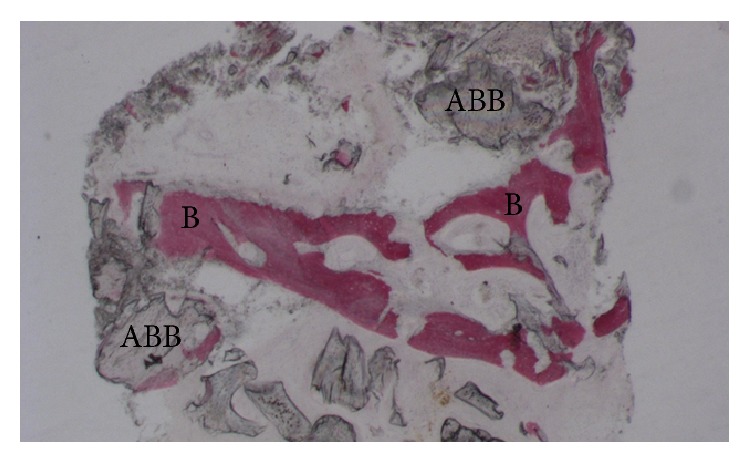
Trabecular bone (B) with large marrow spaces and residual ABB (ABB) particles can be observed (acid fuchsin-toluidine blue; original magnification 30x).

**Figure 4:**
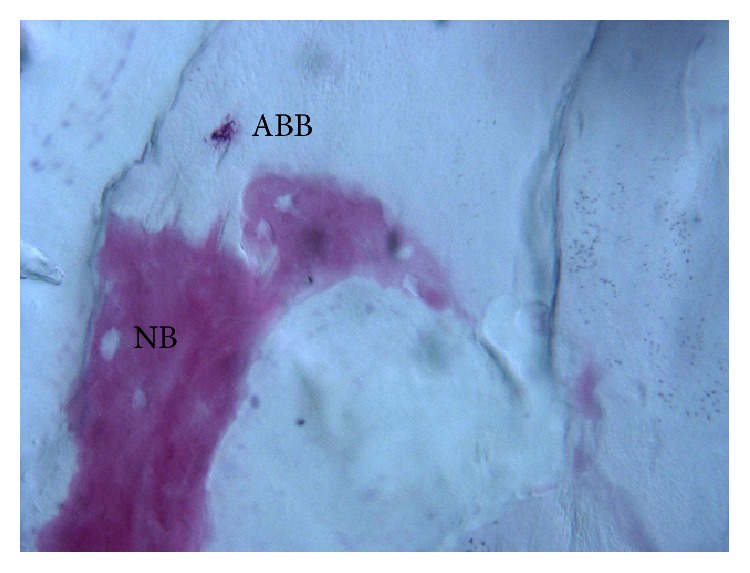
Newly formed trabecular bone (NB) is in close contact with ABB (ABB) particle, with no gaps at the bone-particle interface (acid fuchsin-toluidine blue; original magnification 400x).

At low magnification, trabecular bone with large marrow spaces was observed (Figure 3). Some of the particles appeared to be cemented by this newly formed bone. At higher magnification, the bone presented wide osteocyte lacunae (Figure 4). A limited number of anorganic bovine bone particles were partially covered by connective tissue. No inflammatory cell infiltrate was present around the particles or at the interface with bone. Histomorphometry showed that newly formed bone represented 20.1%, marrow spaces 60.8%, and the residual graft material 19.1%.

#### 3.2.3. Mineralized Solvent-Dehydrated Bone Allograft (MSDBA)

**Figure 5:**
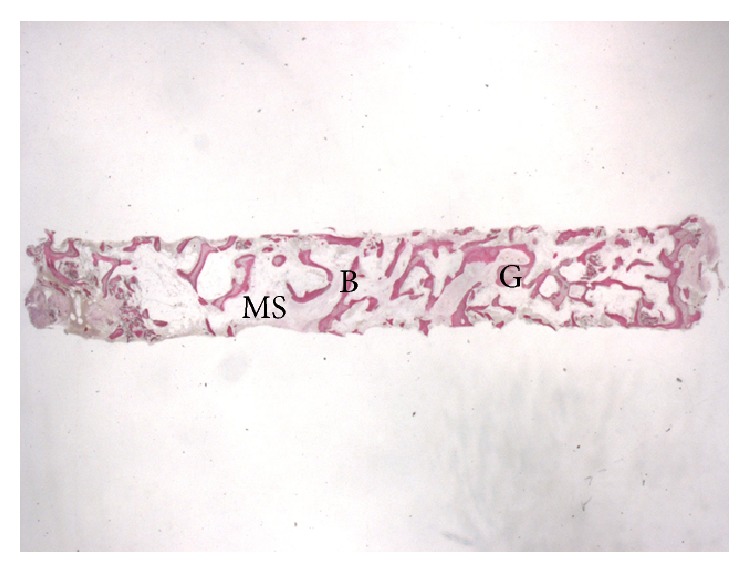
Trabecular bone (B) with large marrow spaces (MS) and residual MSDBA (G) particles can be observed (acid fuchsin-toluidine blue; original magnification 10x).

**Figure 6:**
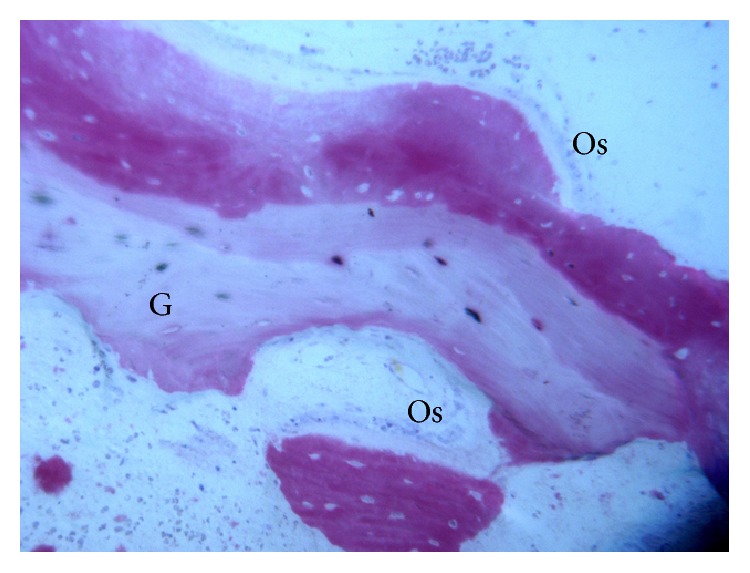
In some fields, osteoblasts (Os) were observed in the process of apposing bone directly on the MSDBA (G) particles' surface (acid fuchsin-toluidine blue; original magnification 200x).

At low magnification, trabecular bone with large marrow spaces was observed (Figure 5). Few particles presented irregularly shaped margins, probably due to a resorption process. There were no gaps at the bone-particle interface and the new bone was in strict contact with the granules. In some fields, osteoblasts were observed in the process of apposing bone directly on the particle surface (Figure 6). Newly formed bone was characterized by large osteocyte lacunae and bridged up most part of the biomaterial particles. Histomorphometry showed that newly formed bone represented 16.4%, marrow spaces 65.1%, and the residual graft material 18.5%.

#### 3.2.4. Equine Bone (EB)

**Figure 7:**
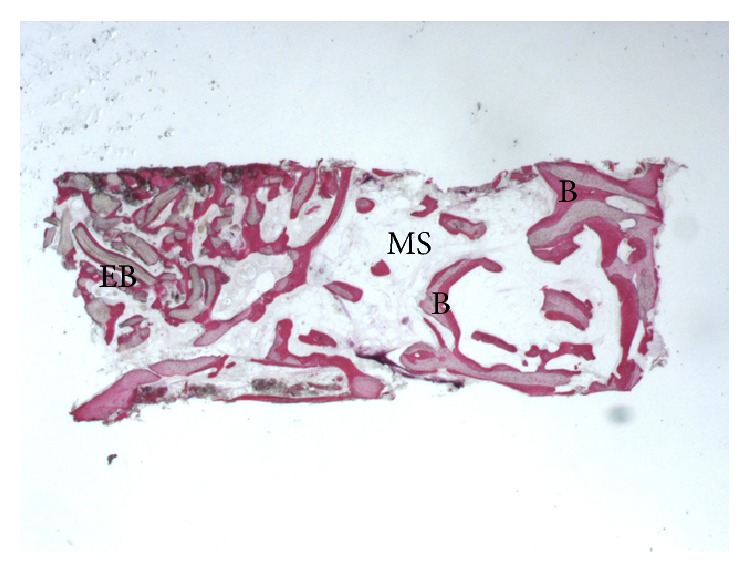
Trabecular bone (B) with large marrow spaces (MS) and residual EB (EB) particles were present (acid fuchsin-toluidine blue; original magnification 12x).

**Figure 8:**
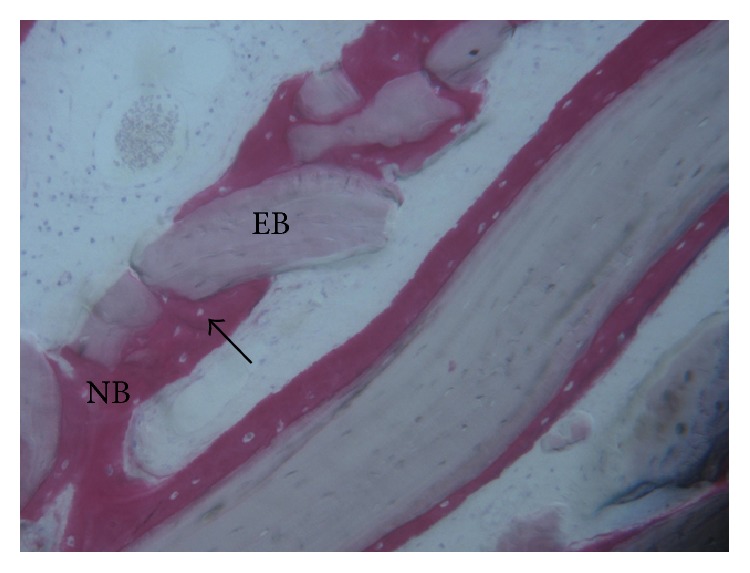
Newly formed bone (NB) with wide osteocyte lacunae (black arrow) in tight contact with EB (EB) particles (acid fuchsin-toluidine blue; original magnification 200x).

At low magnification, trabecular bone with large marrow spaces was observed. In a portion of the specimen, preexisting bone with areas of remodeling was present (Figure 7). Some of the particles appeared to be cemented by the newly formed bone. At higher magnification, the bone showed wide osteocyte lacunae (Figure 8). Some trabeculae of the grafted material were bridged by newly formed bone. No inflammatory cells or multinucleated cells were observed around the particles or at newly formed bone-biomaterial interface in the marrow spaces. Histomorphometry showed that newly formed bone represented 21.9%, marrow spaces 54.9%, and the residual graft material 23.2%.

## 4. Discussion

In sinus augmentation procedures, the choice of bone substitutes plays a key role, because their different properties affect medium and long-term success of implant rehabilitation. However, clinical and histological outcomes about bone substitute materials and management and timing of implant placement and their follow-up still remain open questions, because there are no clear guidelines for the use of autogenous bone or bone substitutes [24]. Numerous studies have compared grafting materials after sinus augmentation, reporting small numbers of histology samples as our investigation [11, 19, 21, 22, 26, 27, 30]. Therefore, it is very important to histologically evaluate the healing process of bone substitute materials, after two-stage sinus augmentation, in order to increase the knowledge about their biological behavior in humans [37].

In the present preliminary investigation, to examine the bone regenerative potential of a newly biphasic calcium phosphate ceramic, the histologic process of healing and bone formation of HA-*β*-TCP (30/70) was compared to those of the most frequently used and well documented biomaterials after six months from sinus augmentation [10–13, 17–20]. The present scaffold was developed by the direct rapid prototyping technique dispense-plotting, as previously described by Deisinger et al. [38] and Mangano et al. [18]. This method enables tuning the HA/*β*-TCP ratio, surface, structure, and micro- and macroporosity which in turn influence the regenerative potential. Indeed, the pattern of resorption and the healing process would be influenced by the variation of the ratio between the component at slow resorption (HA) and those at more fast resorption (*β*-TCP), even if no statistical significant difference was found by Schopper et al. [39], comparing two different ratios (30/70 versus 50/50) of HA/TCP in a sheep model, although a better integration into the host bone was observed in the 30/70 composition. On the contrary in a comparative study of biphasic calcium phosphates with different HA/TCP ratios in mandibular bone defects of minipig, the BCP (20/80), unlike BCP (60/40) and BCP (80/20), showed results similar to those of autogenous bone grafts, indicating that the ratio of HA/TCP (60/40) might not be optimal for bone healing [40]. The three-dimensional network with an interconnecting pore structure promotes the vascularization that is essential for the proliferation and differentiation of osteoblasts and therefore for the ingrowth of new bone into the graft [41].

At the histologic examination, the HA-*β*-TCP (30/70) scaffold showed a pattern of bone formation similar to the other biomaterials tested, with a large number of grafts, completely surrounded by newly formed bone, that was always in close contact, without gaps, with the particles. Furthermore, osteoblasts, in some fields, were detected in the apposing bone directly on the particle surface, the marrow stromal cells and blood vessels were found inside the marrow spaces and the vascular growth was observed next to the newly formed bone. These histological data, in agreement with those reported in a similar study about evaluation of HA-*β*-TCP (40/60) in sinus elevation after a healing period of 6 months [42], demonstrated that the HA-*β*-TCP is an osteoconductive and resorbable material. The absence of inflammatory cells or foreign body reaction around the grafted particles testifies safety and biocompatibility.

For the ABB sample a limited number of grafted particles were found partially covered by connective tissue while some of the particles appeared to be cemented by this newly formed bone. The ABB sample was smaller than the others and this was due to the fracture of the bone biopsy retrieved from the ABB augmented site during the removal from the trephine bur. ABB is a nonresorbable bone substitute and has osteoconductive properties. Its structure could represent a type of protection against bone resorption, guaranteeing long-term stability of the augmented maxillary sinus [10]. The MSDBA sample confirmed the biocompatibility, ease of use, and ability to form and maintain new bone in the maxillary sinus as reported by previous studies [11, 26]. No signs of acute inflammation were present, and the percentage of newly formed bone and residual graft material were comparable to those reported for other biomaterials [43]. The EB sample showed some trabeculae bridged by newly formed bone. Our results are comparable to other investigations [12, 13]. It is evident resorption phenomena, and its ability to achieve a more rapid and intense vascularization is very important also in influencing long-term integration and predictability of implant-prosthetic rehabilitation in regenerated sites. Its higher resorption ability could probably be due to a deantigenation process this grafting material undergoes [13]. The results of the present investigation revealed that HA-*β*-TCP (30/70) could provide a better result in sinus augmentation procedures compared to the other biomaterials. No histological signs of adverse reactions were observed. Results from this preliminary investigation have shown that HA-*β*-TCP (30/70) has good biocompatibility, with no histological signs of adverse reactions, and osteoconductivity, with bone formation directly on the biomaterial surface. Nevertheless the presence of a high quantity of the biomaterial particles after 6 months will require long-term histological studies to better understand the times and the modalities of the resorption of this graft.

In conclusion, within the limitations of the present report the histological and histomorphometric observations support the fact that Ha-TCP (30/70) can be suitable for successful augmentation procedures of the maxillary sinus and represents a very interesting option.
